# Systematic Investigation of *FLOWERING LOCUS T*-Like Poaceae Gene Families Identifies the Short-Day Expressed Flowering Pathway Gene, *TaFT3* in Wheat (*Triticum aestivum* L.)

**DOI:** 10.3389/fpls.2016.00857

**Published:** 2016-06-22

**Authors:** Joanna Halliwell, Philippa Borrill, Anna Gordon, Radoslaw Kowalczyk, Marina L. Pagano, Benedetta Saccomanno, Alison R. Bentley, Cristobal Uauy, James Cockram

**Affiliations:** ^1^Crop Genetics Department, John Innes CentreNorwich, UK; ^2^John Bingham Laboratory, National Institute of Agricultural BotanyCambridge, UK; ^3^Faculty of Life Sciences, University of ManchesterManchester, UK; ^4^Department of Chemical, Biological, Pharmaceutical and Environmental Sciences, University of MessinaMessina, Italy

**Keywords:** homoeolog-specific gene expression, environmental adaptation, quantitative RT-PCR, flowering time

## Abstract

To date, a small number of major flowering time loci have been identified in the related Triticeae crops, bread wheat (*Triticum aestivum*), durum wheat (*T. durum*), and barley (*Hordeum vulgare*). Natural genetic variants at these loci result in major phenotypic changes which have adapted crops to the novel environments encountered during the spread of agriculture. The polyploid nature of bread and durum wheat means that major flowering time loci in which recessive alleles confer adaptive advantage in related diploid species have not been readily identified. One such example is the *PPD-H2* flowering time locus encoded by *FLOWERING LOCUS T 3* (*HvFT3*) in the diploid crop barley, for which recessive mutant alleles confer delayed flowering under short day (SD) photoperiods. In autumn-sown barley, such alleles aid the repression of flowering over the winter, which help prevent the development of cold-sensitive floral organs until the onset of inductive long day (LD) photoperiods the following spring. While the identification of orthologous loci in wheat could provide breeders with alternative mechanisms to fine tune flowering time, systematic identification of wheat orthologs of *HvFT3* has not been reported. Here, we characterize the *FT* gene families in six Poaceae species, identifying novel members in all taxa investigated, as well as *FT3* homoeologs from the A, B and D genomes of hexaploid (*TaFT3*) and tetraploid wheat. Sequence analysis shows *TaFT3* homoeologs display high similarity to the *HvFT3* coding region (95–96%) and predicted protein (96–97%), with conservation of intron/exon structure across the five cereal species investigated. Genetic mapping and comparative analyses in hexaploid and tetraploid wheat find *TaFT3* homoeologs map to the long arms of the group 1 chromosomes, collinear to *HvFT3* in barley and *FT3* orthologs in rice, foxtail millet and brachypodium. Genome-specific expression analyses show *FT3* homoeologs in tetraploid and hexaploid wheat are upregulated under SD photoperiods, but not under LDs, analogous to the expression of *HvFT3*. Collectively, these results indicate that functional wheat orthologs of *HvFT3* have been identified. The molecular resources generated here provide the foundation for engineering a novel major flowering time locus in wheat using forward or reverse genetics approaches.

## Introduction

The rising global human population requires increases in agricultural productivity in order to meet food demand. However, the effects of climate change on crop harvests have the potential to cause wide-scale food shortage and fluctuations in supply. Accordingly, there is a need to develop crop varieties adapted to maintain and increase yields in the face of future climatic change. Given the central role of flowering time in the adaption of crops to local agricultural environments, the efficient manipulation of this trait is of particular interest under future climate change scenarios.

A small number of major flowering time loci have been identified in temperate cereal crops such as barley (*Hordeum vulgare*; diploid, 2*n* = 2x = 14), durum wheat (*Triticum durum*; tetraploid, 2*n* = 4x = 28) and bread wheat (*T. aestivum*; hexaploid, 2*n* = 6x = 42). A subset of these loci are located at collinear chromosomal locations in multiple species, and are encoded by orthologous genes (Cockram et al., 2007a). For example, major photoperiod response loci are located on the short arm of the group 2 chromosomes of barley (*PPD-H1*), rye (*Secale cereale, PPD-R1*), durum wheat (*PPD-A1, -B1*), and bread wheat (*PPD-A1, -B1, -D1*), encoded by orthologous *PSEUDO RESPONSE REGULATOR* (*PRR*) genes (reviewed by Bentley et al., 2013a). While wild-type alleles promote floral transition under LD photoperiods, the naturally occurring (semi-) dominant mutant alleles confer insensitivity to day length (Bentley et al., 2013b). Similarly, (semi-) dominant mutant alleles at collinear cereal *VERNALIZATION 1* (*VRN-1*) loci result in the abolishment of vernalization requirement, resulting in rapid-cycling accessions which can proceed to flowering without the need for vernalization treatment (prolonged periods of low non-freezing temperature) (Cockram et al., 2007a,b). During the domestication of temperate cereals, the dominant nature of such mutations meant the changes to flowering time they confer were readily selectable by Neolithic farmers (Jones et al., 2011). Conversely, phenotypes conferred by recessive mutations would have been harder to select, as they require fixation as recessive-homozygous before a visible phenotype was apparent. As ploidy increases, the higher the number of recessive mutations that must be created, fixed at homozygosity, and combined with homozygous recessive alleles at all other homoeologs. Thus, beneficial traits controlled by recessive alleles would historically have been difficult to select in polyploids (Borrill et al., 2015).

The advent of molecular genetics, however, means that it should now be possible to engineer such loci using forward and/or reverse genetic approaches. This is exemplified by the orthologous *VRN2* vernalization response loci in the Triticeae, which modulate flowering in response to vernalization. In the diploid species barley and *T. monococcum*, recessive alleles at *VRN2* (generated by mutation or deletion of the underlying *ZCCT* genes) abolish vernalization requirement (Yan et al., 2004). However until the advent of molecular genetic approaches, *VRN2* loci had not been identified in polyploid wheat species. Recent studies in *T. durum* show that the sequence-based identification of recessive *VRN2* alleles, in conjunction with allele tracking within crosses between recessive carriers, allow the creation of vernalization insensitive tetraploid wheat by combining recessive *vrn-A2* and *vrn-B2* alleles (Distelfeld et al., 2009).

In barley, the major flowering time locus *PPD-H2* is thought to be encoded by the *FLOWERING LOCUS T* (*FT*)-like gene *HvFT3* (Faure et al., 2007; Kikuchi et al., 2009), with recessive alleles conferring delayed flowering under SD photoperiods. Allelic variation at *PPD-H2* is a key component of local adaptation, with winter and spring varieties showing an almost perfect partition between mutant and wild type alleles, respectively (Cockram et al., 2015). While orthologous genetic loci have not been identified at collinear locations in the wheat genome, this may be due to its polyploid nature masking phenotypic effect. If *HvFT3* orthologs are present (and expressed) in wheat, the identification and consolidation of recessive alleles at all three homoeologous genes into one genetic background may provide a novel source of flowering time variation of particular relevance to a warming climate, allowing floral repression in the winter without the need for a strong vernalization requirement. Indeed, “alternative” barley varieties (cold hardy spring types that lack a vernalization requirement but can be planted in the autumn for harvest the following summer) appear to possess flowering time gene haplotypes that invariably include recessive *ppd-H2* alleles, thus helping to prevent premature flowering (Cockram et al., 2015). To date, no wheat *FT3* orthologs have been identified within the extensive expressed sequence tag (EST) databases (and only six barley ESTs were present prior to specific investigation of *HvFT3*). However, the recent availability of survey sequence of the bread wheat genome offers new opportunities for the investigation of wheat gene content. This provides a timely opportunity to use reverse genetic approaches to create a wheat accession possessing a SD floral delay analogous to that deployed in winter barley, and of particular relevance to climate change scenarios.

Here, we systematically identify orthologs of *HvFT3* in the A, B, and D genomes of hexaploid and tetraploid wheat, show that they (i) group phylogenetically with *FT3* genes from barley and other cereals, (ii) map to collinear positions in the related cereal species wheat, barley, rice (*Oryza sativa*) and *Brachypodium distachyon*, and (iii) are upregulated under SD photoperiods. Collectively, these resources provide the foundation from which forward and reverse genetic approaches can be deployed toward the creation of novel SD responsive flowering time loci in polyploid wheat, aiding adaptation to future climate change scenarios.

## Materials and methods

### Bioinformatic and phylogenetic analysis

Barley *HvFT3* coding regions (CDS, GenBank accession HM133572) were used for BLASTn analysis (match / mismatch scores = 2, 3; gap costs: existence = 5, extension = 2, expectation (*e*)-value threshold of 1.0e-30) to identify putative orthologs in the genomic survey sequence of bread wheat cv. Chinese Spring (The International Wheat Genome Sequencing Consortium, 2014; assembly IWGSC1.0+popseq) and barley cv. Morex (The International Barley Genome Sequencing Consortium, 2012; assembly ASM32608v1, INSDC Assembly GCA_000326085.1, Mar 2012), as well as in the sequenced genomes of *Brachypodium distachyon* L. accession Bd21 (The International Brachypodium Genome Sequencing Consortium, 2010; assembly v1.0), sorghum (*Sorghum bicolour* accession BTx623) (Paterson et al., 2009; v1.0), and foxtail millet (*Setaria italica* accession Yugu1) (Zhang et al., 2012; assembly v1.0). *De novo* gene predictions were performed using FGENESH (http://www.softberry.com/). The previously determined rice *FTL* gene family is as described by Higgins et al. (2010), as are brachypodium and barley *FT1* through to *FT7* genes. For comparative analyses, colinearity was determined via BLASTn using CDS as queries and with an *e*-value significance threshold <e-4. Macro-colinearity between cereal genomes is as previously described (Devos, 2005; Paterson et al., 2009; International Brachypodium Initiative, 2010). Wheat *FT* gene nomenclature follows recommended rules for gene symbolization (http://wheat.pw.usda.gov/GG2/Triticum/wgc/2008/).

Protein domains were determined using Pfam v25.0 (Finn et al., 2010) and Prosite v20.79 (http://prosite.expasy.org/). Predicted protein sequences were aligned using Clustal Omega (Sievers et al., 2011) and manually edited using GENEDOC v2.6 (http://www.nrbsc.org/gfx/genedoc/). The resulting alignments were used for phylogenetic analysis, conducted using the PHYLIP package v3.5 (Felsenstein, 1989). Unrooted phylogenies were determined using distance matrix, with tree topographies supported by bootstrapping (1000 replicates). Predicted gene and protein models are prefixed with the genus and species initials throughout. DNA sequences from DArT markers were accessed via http://www.diversityarrays.com/dart-map-sequences.

### DNA extraction, primer design, and PCR

Genomic DNA was extracted from wheat leaf material using the DNeasy 96 Plant Kit (Qiagen). Primers were designed from genomic DNA sequence contigs using Primer3 v0.4.0 (http://primer3.sourceforge.net/), with pairs selected based on the number of homoeolog-specific nucleotides incorporated. Polymerase chain reaction (PCR) amplification (10 μl reactions) were performed using the reagents shipped in the FastStart *Taq* DNA polymerase kit (Roche). PCR cycling was carried out using a Veriti 96 well Thermo Cycler (Applied Biosystems) with the parameters: 5 min at 96°C, followed by 35 cycles of 50 sec at 96°C, 50 sec annealing temperature, 90 sec at 72°C, final extension of 7 min at 72°C. Primer sequences and annealing temperatures are listed in Table 1.

**Table 1:** **Genome-specific ***TaFT3*** primers used for genetic mapping and DNA sequencing**.

**Target gene**	**Annealing temperature (°C)**	**Primer sequence (5′ → 3′)**
*TaFT3-A1*	62	F1: GCCCGACCACTCCATAAAGTA
		R1: GGTGTTAACGATGGGCTATATTA
*TaFT3-B1*	61	F2: GTATACCACAGCCATGCTAATG
		R2: AGATATCTTGGTGTTAATGATGGAC
*TaFT3-D1*	62	F1: CGCCCACAATTCACAAGTTT
		R1: ATTCTTTGTCATGGTTCAAGATG
*TaFT3-D1*	64	F4: ATCGTTAACACCAAGACATCCTG
		R4: TGTTGATTTATTCAATAGAACTTGATG

*Annealing temperatures used each homoeolog-specific primer pair during PCR are indicated*.

### Genotyping and genetic mapping

Genetic mapping was undertaken in hexaploid wheat using the Robigus x Solstice population consisting of 200 doubled haploid (DH) lines (Gordon et al., 2015), and selected due to the relevance of the parents in modern European wheat pedigrees. *TaFT3* sequencing in parental lines used a minimum of three independent PCRs as templates. Sequencing was conducted using BigDye kit v3.1 (Applied Biosystems), following previously described protocols (Mameaux et al., 2012). Sequence traces were edited and manipulated using the VectorNTI Advance package v10.1.1 (Invitrogen, Paisley, UK). DNA polymorphisms for *TaFT3-A1* was genotyped in the mapping population by direct sequencing of PCR amplicons using the polymorphisms listed in Table 1. The *TaFT3-B1* presence/absence polymorphism was genotyped in duplicate in the bi-parental mapping population via electrophoresis on 1% agarose gels, and visualized with ethidium bromide staining under UV light. Genetic mapping was conducted by combining the *TaFT3* genotype data generated here along with a genome-wide marker set described by Gordon et al. (2015). Genetic maps were created using MapDisto version 1.7.5 Beta 4 (Lorieux, 2012) using the Haldane mapping function with a maximum recombination fraction of 0.3 and LOD of 6.

### Plant growth and tissues

Two hexaploid (Chinese Spring, Cadenza) and one tetraploid (Kronos) spring wheat varieties were used for gene expression analyses. Varieties were selected for the following reasons: (1) Chinese Spring: worldwide wheat reference variety for genome sequencing. (2) Cadenza: parent of the UK reference Avalon × Cadenza genetic mapping population; Ethyl methanesulfonate (EMS) mutated Targeting Induced Local Lesions in Genomes (TILLING) population available (Rakszegi et al., 2010). (3) Kronos: EMS mutated TILLING population available (Uauy et al., 2009). Seeds were planted into cereal compost filled P2 pots and grown under LD (16 h light/8 h dark) and SD (8 h light/16 h dark) photoperiods in a growth cabinet (Sanyo) with day/night temperatures of 20/10°C. For each of the two photoperiod treatments, tissue samples (all of the aerial plant) were harvested at 1 week intervals post emergence for a total of 4 weeks. Six biological repetitions were harvested for every variety at each time point. In addition, a week 0 time point was taken by sampling tissue from 7 to 10 wheat embryos after imbibing for 2 days at 4°C. All tissue was frozen in liquid nitrogen immediately after harvesting, and subsequently stored at −80°C. Shoot apical meristems were dissected from seedlings at 2 and 4 weeks using a stereomicroscope, and imaged using a digital camera mounted to a stereomicroscope.

### RNA extraction, cDNA synthesis, and quantitative RT-PCR

Plant tissue was ground under liquid nitrogen using a pestle and mortar, and RNA extracted using Tri-reagent (Sigma), following the manufacturer's instructions. RNA concentrations were determined using a Nanodrop 2000 spectrometer (Thermo Fischer Scientific), standardized to 250 ng/μl using nuclease-free water (Promega), and RNA samples (1.75 μg each) were DNAse treated using a RQ1 RNase-Free DNAse kit (Promega). Prior to cDNA synthesis, genomic DNA was digested by addition of RNase-free DNase (Promega) to each sample, and reactions terminated using RQ1 DNase Stop Solution (Promega), following the manufacturer's instructions. cDNA synthesis performed using a M-MLV Reverse Transcriptase kit (Promega), according to the manufacturer's instructions. All qRT-PCR reactions were undertaken using SYBR MM (Roche), and carried out in total reaction volumes of 10 μl using a Light Cycler 480 (Roche) with the following cycling conditions: 95°C 5 mins; 45 cycles of 95°C for 10 s, 60°C for 15 s, 72°C for 30 s, cooling 60°C; melt curve was carried out by ramping 0.11°C/s from 60 to 95°C, with 5 readings taken per second. RT-PCR primers for the three *TaFT3* homoeologs and the four reference genes (*ACTIN, UBIQUITIN, GAPDH, EF1A*) are listed in Supplementary Table 1. Primer efficiencies were based on a 1:2 cDNA dilution series. Reaction volumes of 10 μl were used, with final cDNA concentrations ranging from 0.013 to 2.08 ng μl^−1^. Three technical repeats were undertaken for each primer pair. Primer efficiencies were calculated using the formula: EFFICIENCY = −1+10(−1/Gradient), where Gradient = LINEST (Cp-values; log concentration). The relative expression level of *TaFT3* compared to the four reference genes was calculated using the Pfaffl method (Pfaffl, 2001), where the reference gene efficiency (denominator) was the normalization factor calculated by Genorm across all four reference genes (Vandesompele et al., 2002).

## Results

### *FT3* genes in wheat

**Figure 1:**
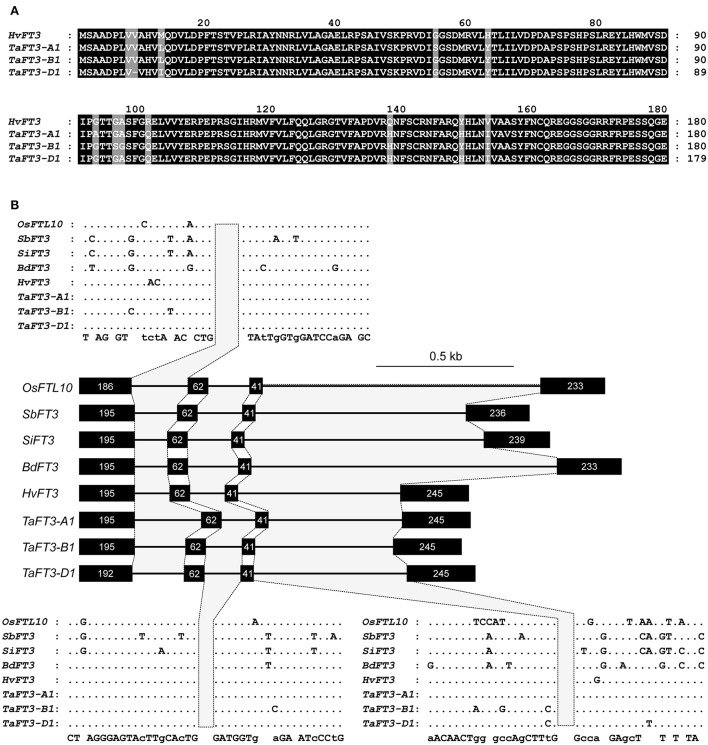
**Cross-species comparison of ***FT3*** proteins and gene structure. (A)** Alignment of predicted *FT3* proteins from wheat (UniProtKB accessions W4ZP37, W5AOP4, W5AIL5) and barley (GenBank accession ADW83188). **(B)** Intron/exon structure of *FT3* genes from six Poaceae species. Nucleotide number per exon is indicated within each exon. *SbFT3* was not included, as compared to all other *FT* genes, the gene model is truncated at the 5′ end.

To identify wheat homologs of the *HvFT3* gene thought to underlie the barley *PPD-H2* locus, the barley *HvFT3* CDS (HM133572) was used for BLASTn searches of hexaploid wheat sequence contigs in the flow sorted wheat genome survey sequence, identifying 21 significant (<e −5) hits. Of these, the three highest hits (*e* = 0, percentage identity >80%) all originated from DNA sequenced from the long arm of the group 1 chromosomes (Supplementary Table 2), colinear with barley chromosome 1H, to which *HvFT3* has been genetically mapped (Faure et al., 2007; Kikuchi et al., 2009). Gene prediction identified full length *FT3*-like genes composed of four exons on all three contigs, with predicted CDS of 543 bp (*TaFT3-A1, TaFT3-B1*) and 540 bp (*TaFT3-D1*) (Supplementary Table 2). Back-BLASTn analysis of the identified *TaFT3* CDS to the draft barley genome sequence identified a genomic region on the long arm of chromosome 1H (1:419425259-419425459; morex_contig_2551337) as the best hit (<3.8e-78, percentage identity >95%), on which *HvFT3* is located based on BLASTn analysis using barley *HvFT3* CDS. Wheat *FT3* genes from the A, B, and D genomes were found to show high similarity to *HvFT3* at the cDNA (95, 95, and 96%, respectively) and protein (96, 97, and 97%) levels (Figure 1). Intron/exon structure was also highly conserved, with all five cereal species investigated predicted to possess four introns, with intron/exon boundaries located at conserved positions (Figure 1). TaFT3 predicted proteins encoded by all three homoeologs contained the conserved Phosphatidylethanolamine-binding protein (PEBP) domain present in barley HvFT3.

### Poaceae *FT* genes

To further characterize the wheat *TaFT3* genes within the context of the wider Poaceae *FT* gene families, 12 of the 13 rice *FTL* genes were used for BLASTn searches of the sequenced cereal species brachypodium, sorghum, foxtail millet, as well as the draft genome assemblies of barley and wheat (Table 2). *OsFTL11* was not included due to its previously reported differentiation from true *FT*-gene family (Faure et al., 2007). Homologs were identified in all species for nine rice genes (*OsFTL1, OsFTL2, OsFTL4, OsFTL5, OsFTL6, OsFTL9, OsFTL10, OsFTL12*, and *OsFTL13*), while homoeologs for *OsFTL7* were identified in all species apart from brachypodium. In some instances, multiple homologs were identified within a species: for *OsFTL2*, three homologs were identified in foxtail millet (*SiFT1A, SiFT1B, SiFT1C*), while for *OsFTL12*, two homologs were identified on the wheat A genome (*TaFT4-A1, TaFT4-A2*). *OsFTL10* identified at least two full length homoeologs on each of the three genomes of bread wheat (termed here *TaFT5-A1, -A2, TaFT5-B1, -B2, TaFT5-D1, -D2, -D4*), with an additional third truncated copy on the D genome (*TaFT5-D3*). For *OsFTL13*, two homologous genes were identified in sorghum and foxtail millet (classified *SbFT6A*/*SbFT6B* and *SiFT6A*/*SiFT6B*, respectively), as well as on the wheat D genome (*TaFT6-D1, TaFT6-D2*). Additional *FT*-like genes were found in the temperate grass species (wheat, barley, and brachypodium) that do not have homologous genes in the tropical grass species investigated (rice, sorghum, and foxtail millet). Here, these have been termed the *FT8* and *FT11* genes, with the former compromising of multiple copies, in barley (*HvFT8A, HvFT8B, HvFT8C*), brachypodium (*BdFT8A, BdFT8B, BdFT8C*) and wheat (*TaFT8-A1, -A2, -B1, -B2, -B3, -B4, -D1*). No homologs were identified for *OsFLT8*. *OsFTL3* represents a tandem duplication of *OsFTL2*, unique to rice among the species investigated.

**Table 2:** *****FT*** genes in rice, sorghum, foxtail millet, brachypodium, barley, and bread wheat**.

**Gene**	**Chr (Mbp)**	**Gene model^a^**	**Exons**	**Protein (aa)**	**Closest rice *FTL* homolog**	**Homology to rice gene: e-value (% identity)^c^**
***O. sativa***						
*OsFTL1*	1 (6.50)	Os01g11940.1	4	276	N/A	N/A
*OsFTL2*	6 (2.95)	Os06g06320.1	4	179	N/A	N/A
*OsFTL3*	6 (2.93)	Os06g06300.1	4	178	N/A	N/A
*OsFTL4*	9 (19.98)	Os09g33850.1	4	173	N/A	N/A
*OsFTL5*	2 (23.59)	Os02g39064.1	4	174	N/A	N/A
*OsFTL6*	4 (24.39)	Os04g41130.1	4	174	N/A	N/A
*OsFTL7*	12 (7.24)	Os12g13030.1	4	177	N/A	N/A
*OsFTL8*	1 (5.65)	Os01g10590.1	4	169	N/A	N/A
*OsFTL9*	1 (31.34)	Os01g54490.1	4	175	N/A	N/A
*OsFTL10*	5 (25.67)	Os05g44180.1	4	174	N/A	N/A
*OsFTL11*	11 (10.74)	Os11g18870.1	4	174	N/A	N/A
*OsFTL12*	6 (20.97)	Os06g35940.1	4	173	N/A	N/A
*OsFTL13*	2 (7.49)	Os02g13830.1	4	185	N/A	N/A
***S. bicolor***						
*SbFT1*	10 (3.46)	Sb10g003940.1	4	179	*OsFTL2*	4e-68 (91.1)
*SbFT2*	3 (1.51)	FGENESH00000046519	**5**	173	*OsFTL1*	2e-64 (88.9)
SbFT3^*^	9 (55.15)	Sb09g025760.1^*^	4^*^	118^*^	*OsFTL10*^l^	6e-18 (86.5)
*SbFT4*	10 (48.39)	Sb10g021790.1	4	173	*OsFTL12*	5e-43 (86.8)
*SbFT5*	3 (62.75)	Sb03g034580.1	4	177	*OsFTL9*^l^	1e-37 (83.9)
*SbFT6A*	4 (9.46)	Sb04g008320.1	4	182	*OsFTL13*	1e-77 (91.5)
*SbFT6B*	6 (33.53)	Sb06g012260.1	4	185	*OsFTL13*	8e-42 (86.0)
*SbFT7*	8 (15.21)	Sb08g008180.1	4	177	*OsFTL7*	1e-80 (92.3)
*SbFT9*	6 (50.27)	Sb06g020850.1	4	174	*OsFTL6*	2e-51 (86.2)
*SbFT10*	2 (64.81)	Sb02g029725.1	4	173	*OsFTL4*	2e-73 (92.5)
*SbFT11*	unknown	Sb0010s003120.1	4	174	*OsFTL11*	1e-31 (83.7)
*SbFT12*	4 (55.04)	Sb04g025210.1	4	174	*OsFTL5*	2e-66 (91.3)
***S. italica***						
*SiFT1A*	4 (5.21)	Si008517m.g	4	178	*OsFTL2*	3e-84 (92.2)
*SiFT1B*	4 (12.52)	Si007366m.g	4	177	*OsFTL2*	3e-66 (90.4)
*SiFT1C*	4 (9.10)	Si008120m.g	4	165	*OsFTL2*	3e-38 (86.7)
*SiFT2*	5 (13.59)	*De novo* prediction^b^	4	173	*OsFTL1*	4e-62 (89.2)
*SiFT3*	3 (13.16)	*De novo* prediction^b^	4	199	*OsFTL10*^k^	2e-27 (82.3)
*SiFT4*	4 (29.10)	Si007376m.g	4	173	*OsFTL12*	7e-39 (85.2)
*SiFT5*	5 (36.83)	Si005012m.g	4	179	*OsFTL9*	3e-41 (86.3)
*SiFT6A*	1 (2.32)	Si020173m.g	4	184	*OsFTL13*	6e-64 (88.8)
*SiFT6B*	7 (17.29)	Si011866m.g	4	184	*OsFTL13*	1e-52 (87.8)
*SiFT7*	3 (5.97)	Si025116m.g	3	175	*OsFTL7*	3e-90 (94.1)
*SiFT9*	7 (23.45)	Si012372m.g	4	174	*OsFTL6*	3e-56 (87.0)
*SiFT10*	2 (37.13)	*De novo* prediction^b^	4	173	*OsFTL4*	2e-67 (89.6)
*SiFT11*	8 (14.74)	Si027589m.g	4	174	*OsFTL11*	5e-43 (85.9)
*SiFT12*	1 (30.15)	Si020105m.g	4	174	*OsFTL5*	6e-61 (87.9)
***B. distachyon***						
*BdFT1*	1 (47.49)	Bradi1g48830.1	4	177	*OsFTL2*	2e-76 (90.8)
*BdFT2*	2 (5.40)	Bradi2g07070.1	4	173	*OsFTL1*	2e-71 (90.2)
*BdFT3*	2 (17.34)	Bradi2g19670.1	4	180	*OsFTL10*	4e-28 (83.3)
*BdFT4*	1 (34.32)	Bradi1g38150.1	4	173	*OsFTL12*	2e-57 (88.5)
*BdFT5*	2 (49.83)	Bradi2g49795.1	4	177	*OsFTL9*	2e-29 (84.7)
*BdFT6*	3 (7.00)	Bradi3g08890.1	4	182	*OsFTL13*	1e-58 (89.2)
*BdFT8A*	4 (44.37)	Bradi4g39750.1	4	171	*OsFTL7*	1e-55 (87.9)
*BdFT8B*	4 (44.37)	Bradi4g39760.1	4	172	*OsFTL7*	3e-69 (90.9)
*BdFT8C*	4 (44.36)	Bradi4g39730.1	4	173	*OsFTL7*	2e-66 (89.8)
*BdFT9*	5 (17.44)	Bradi5g14010.1	4	174	*OsFTL6*	2e-64 (88.7)
*BdFT10*	4 (40.55)	Bradi4g35040.1	4	173	*OsFTL4*	1e-86 (93.2)
*BdFT12*	3 (49.57)	Bradi3g48036.1	4	174	*OsFTL5*	2e-66 (91.3)
***H. vulgare***						
*HvFT1*	7H (41.48)	MLOC_68576.1	3	177	*OsFTL2*	7e-73 (90.9)
*HvFT2*	3H (45.99)^h^	DQ297407	4	178	*OsFTL1*	2e-70 (90.4)
*HvFT3*	1H (419.42)^i^	HM133572	4	180	*OsFTL9*^k^	9e-20 (81.2)
*HvFT4*	2H (71.03)	MLOC_74854.1	4	173	*OsFTL12*	1e-40 (85.0)
*HvFT5*	4H (2.99)^j^	EF012202.1	4	180	*OsFTL10*	1e-15 (93.2)
*HvFT6*	6H (175.57)	Morex_contig_54196^d^	4	182	*OsFTL13*	6e-61 (88.3)
*HvFT7*	5H (302.12)	Morex_contig_1573409^e^	4	178	*OsFTL7*	2e-67 (90.2)
*HvFT8A*	2H (601.97)	Morex_contig_37453^d^	4	173	*OsFTL7*	1e-49 (86.4)
*HvFT8B*	2H (609.86)	Morex_contig_1560712^e^	4	173	*OsFTL7*	1e-49 (86.4)
*HvFT8C*	2H (589.61)	Morex_contig_158449^e^	4	172	*OsFTL7*	3e-47 (86.1)
*HvFT9*	2H (390.11)	MLOC_58552.3	4	174	*OsFTL6*	2e-70 (90.0)
*HvFT10*	5H (457.04)	Morex_contig_44860^b^	4	172	*OsFTL4*	2e-79 (91.9)
*HvFT11*	4H (155.44)	MLOC_57326.1	4	179	*OsFTL11*	1e-24 (82.0)
*HvFT12*	6H (245.55)	MLOC_64619.2	4	174	*OsFTL5*	4e-68 (89.2)
***T. aestivum***^m^						
*TaFT1-A1*	7A	Traes_7AS_EBD5F1F54.1	3	177	*OsFTL2*	6e-61 (88.6)
TaFT1-B1^*^	7B	Traes_7BS_581AA844D.1^*^	3^*^	148^*^	*OsFTL2*	2e-42 (86.7)
TaFT1-D1^*^	7D	Traes_7DS_12C14942B.1^*^	3^*^	155^*^	*OsFTL2*	6e-42 (88.7)
TaFT2-A1^*^	3A	Traes_3AS_6D1315D0A.3^*^	3^*^	94^*^	*OsFTL1*	5e-63 (92.5)
*TaFT2-B1*	3B	Traes_3B_2A454DB62.1	2^*^	102^*^	*OsFTL1*	4e-82 (92.7)
*TaFT3-A1*	1A	Traes_1AL_4F90FEB36.1	4	180	*OsFTL10*	1e-25 (82.8)
*TaFT3-B1*	1B	Traes_1BL_2C43B822A.1	4	180	*OsFTL9*	3e-29 (83.1)
*TaFT3-D1*	1D	Traes_1DL_CE737E359.1	4	179	*OsFTL10*	2e-30 (85.1)
*TaFT4-A1*	2A	Traes_2AS_64063A59B.1	4	173	*OsFTL12*	3e-50 (86.9)
*TaFT4-A2*	7A	7AL_4533105^f^	4	171	*OsFTL12*	3e-41 (86.1)
*TaFT4-B1*	2B	Traes_2BS_43A8E1EC8.1	4	173	*OsFTL12*	8e-48 (86.4)
*TaFT4-D1*	2D	Traes_2DS_81D62E5E9.1	4	173	*OsFTL12*	5e-49 (87.2)
*TaFT5-A1*	5A	Traes_5AL_20DFB725B.1	4	178	*OsFTL10*	1e-25 (84.1)
*TaFT5-A2*	5A	5AL_2805675^d^	6	189	*OsFTL10*	3e-26 (83.1)
*TaFT5-B1*	4B	Traes_4BL_EBE908323.1	4	180	*OsFTL10*	2e-26 (83.1)
TaFT5-B2^*^	4B	4BL_6996269^*^^g^	2^*^	133^*^	*OsFTL10*	6e-19 (81.2)
*TaFT5-D1*	4D	Traes_4DL_C4A99BB83.1	4	179	*OsFTL10*	2e-26 (83.1)
*TaFT5-D2*	4D	Traes_4DL_CF206DAA3.1	4	180	*OsFTL10*	6e-24 (82.5)
TaFT5-D3^*^	4D	4DL_14405941^*^^g^	2^*^	133^*^	*OsFTL10*	2e-07 (82.8)
*TaFT5-D4*	4D	Traes_4DL_FC267A763.1	4	180	*OsFTL10*	3e-16 (81.9)
*TaFT6-A1*	6A	Traes_6AS_B3C246E08.1	4	183	*OsFTL13*	2e-70 (90.1)
TaFT6-B1^*^	6B	6BS_1200904^*^^f^	3^*^	104^*^	*OsFTL12*	5e-14 (91.7)
*TaFT6-D1*	6D	Traes_6DS_00E3E39411.1	4	183	*OsFTL13*	1e-77 (90.1)
*TaFT6-D2*	6D	Traes_6DS_00E3E3941.1	4	183	*OsFTL13*	1e-77 (90.1)
TaFT7-A1^*^	5A	Traes_5AL_1AF8FD33F.1^*^	4^*^	156^*^	*OsFTL7*	2e-20 (98.2)
*TaFT7-B1*	5B	Traes_5BL_9A27C4A75.1	4	178	*OsFTL7*	2e-60 (88.8)
TaFT7-D1^*^	5D	Traes_5DL_53D0B5EED.1^*^	4^*^	129^*^	*OsFTL7*	9e-20 (82.4)
*TaFT8-A1*	2A	Traes_2AL_552AE18AD.1	4	173	*OsFTL7*	2e-11 (88.2)
*TaFT8-A2*	2A	Traes_2AL_2F198B97C.1	4	171	*OsFTL7*	2e-36 (84.3)
*TaFT8-B1*	2B	Traes_2BL_879586172.1	4	172	*OsFTL7*	3e-47 (86.1)
*TaFT8-B2*	2B	Traes_2BL_2A9A178A1.1	4	164	*OsFTL7*	1e-49 (88.0)
*TaFT8-B3*	3B	TRAES3BF053100340CFD_t1	4	175	*OsFTL7*	3e-41 (85.1)
*TaFT8-B4*	3B	Traes_25121009B.1	4	175	*OsFTL7*	5e-20 (86.5)
TaFT8-D1^*^	2D	Traes_2DL_16EDF0CD2^*^	1^*^	65^*^	*OsFTL7*	7e-49 (87.6)
*TaFT9-A1*	2A	2AL_6350768^f^	4	174	*OsFTL6*	4e-68 (89.2)
*TaFT9-B1*	2B	Traes_2BL_8DB6CE516.1	4	174	*OsFTL6*	1e-65 (88.7)
*TaFT9-D1*	2D	Traes_2DL_485183B12.1	4	174	*OsFTL6*	2e-63 (88.3)
*TaFT10-A1*	5A	Traes_5AL_EFB6E50C9.2	5	178	*OsFTL4*	2e-85 (93.2)
*TaFT10-B1*	5B	Traes_5BL_52911E1E4.2	4	173	*OsFTL4*	2e-82 (92.7)
TaFT10-D1^*^	5D	5DL_4544439^*^^d^	2^*^	73^*^	*OsFTL4*	2e-65 (91.9)
*TaFT11-A1*	4A	Traes_4AL_68B60F6AA.1	4	179	*OsFTL11*	1e-40 (84.8)
*TaFT11-B1*	4B	Traes_4BS_DCCAE937D.1	4	179	*OsFTL11*	2e-33 (83.4)
*TaFT11-D1*	4D	Traes_4DS_2D08DBB36.1	4	179	*OsFTL11*	1e-40 (84.8)
*TaFT12-A1*	6A	Traes_6AL_66B24F155.1	4	174	*OsFTL5*	1e-65 (88.7)
*TaFT12-B1*	6B	Traes_6BL_DA76A4429.1	4	174	*OsFTL5*	2e-66 (91.3)
*TaFT12-D1*	6D	Traes_6DL_62A8C29E0.1	4	174	*OsFTL5*	4e-65 (91.2)

*No BdFT7 gene was identified in B. distachyon accession BD21. No SbFT8 gene was identified in S. bicolour accession BTx623*.

* *Partially truncated gene/pseudogene*.

a *For foxtail millet, barley and wheat, genomic contigs accession numbers are listed where no previously annotated gene models existed. In these cases, de novo gene models were made via FGENESH, using the species parameters listed below*.

b Manually edited gene prediction. FGENESH gene prediction using the following parameters:

c *rice model*.

d *monocot model*.

e *barley model*.

f *bread wheat model*.

g B. distachyon model. Chromosomal Mb position determined via DNA homology with:

h *Morex_contig_136243*.

i *Morex_contig_2551337*.

j *Morex_contig_1572474*.

k *E-value hits for OsFTL10 marginally lower than to OsFTL9. However, the length of alignment is longer for OsFTL10 compared to OsFTL9, and so OsFTL10 is listed in the table*.

l *While the highest BLASTn hits for OsFT9 and OsFT10 in sorghum are SbFT3 and SbFT5, respectively, investigation of wider colinearity finds the orthologous relationships to be OsFLT9-SbFT5 and OsFTL10-SbFT3 (see Figure 4; Paterson et al., 2009)*.

m *For wheat, PGSB/MIPS version 2.2 gene models are listed, where available. Note, OsFTL11 and its homologs were not included in the analysis due to its previously reported differentiation from the true FT-gene family (Faure et al., 2007)*.

### Phylogenetic analysis

**Figure 2:**
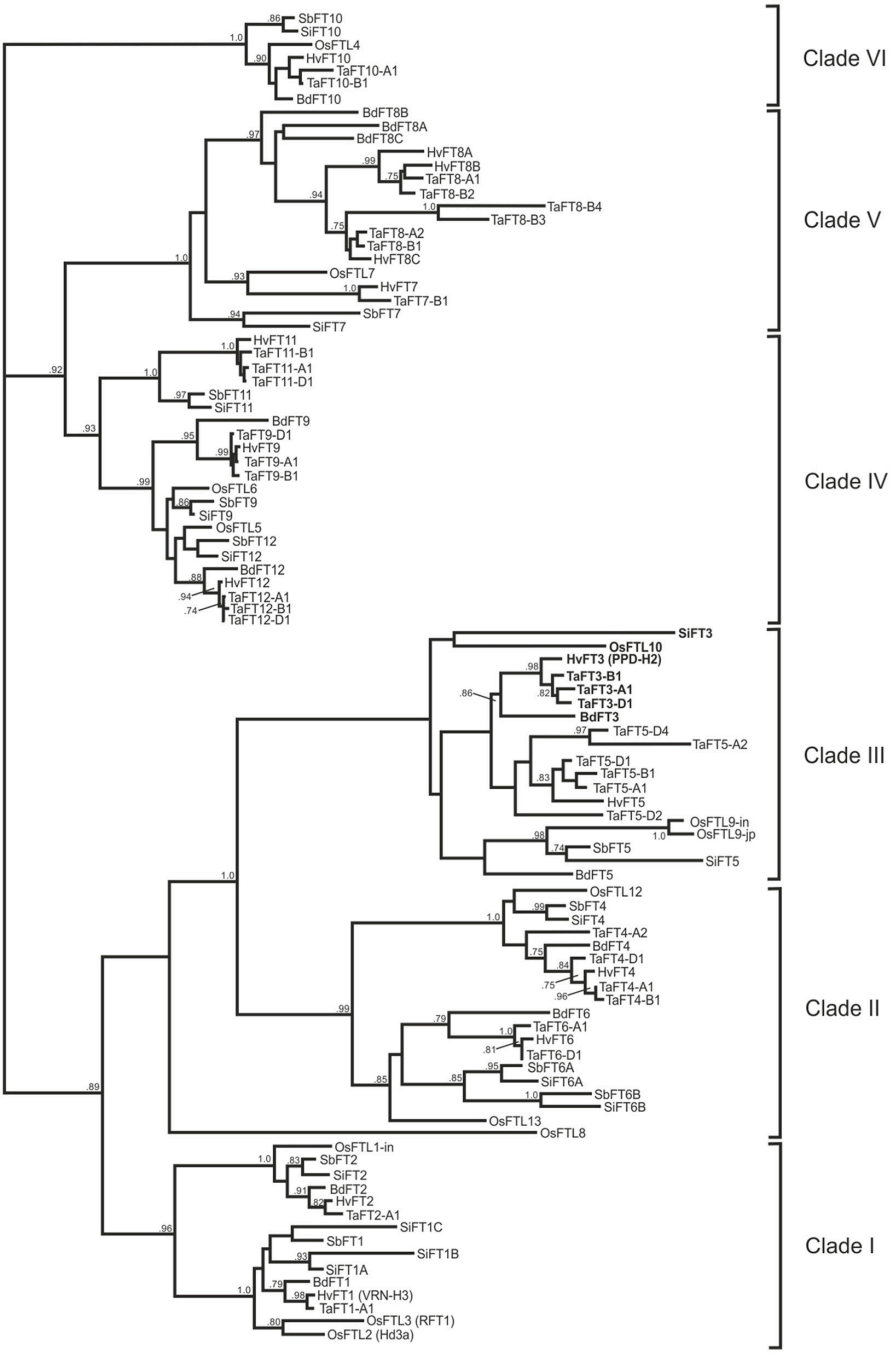
**Phylogenetic analysis of FT predicted proteins from six Poaceae species**. Full-length predicted proteins from rice, sorghum, foxtail millet, brachypodium, barley, and wheat are included (corresponding contig/gene model information listed in Table 1). FT3-like proteins are highlighted in bold. Bootstrap proportions (1000 replicates) ≥0.70 are indicated.

Predicted full length FT protein sequences from rice, brachypodium, sorghum, foxtail millet, and bread wheat were aligned for subsequent construction of a phylogenetic tree. In total, 99 FT-like proteins were used for alignments, resulting in a tree with six broad clades (Figure 2). The first three clades each consisted of two sub-clades, with Poaceae homologs within each sub-group clustering with a rice FTL predicted protein: Clade I (OsFTL1 and OsFTL2/OsFTL3), Clade II (OsFTL12 and OsFTL13), Clade III (OsFTL9 and OsFTL10). Within Clade III, wheat TaFT3 proteins grouped closely with homologs from barley and brachypodium, while homologs from the tropical grass species clustered together with *OsFLT10* in a more disparate group. Clade IV possessed three subclades: while sub-clades 1 and 2 grouped homologs of OsFTL5 and OsFTL6 respectively, the third encompassed the FT11 group of homologs, present in all species except rice. Clade V was composed of two subclades, with homologs of OsFTL7 grouping in one, and temperate cereal specific FT8 proteins grouped in the second. Finally, Clade VI was composed of a single group of proteins homologous to OsFTL4, composing members from all Poaceae species investigated. OsFTL8 was not found to be grouped closely with any clade, but was most similar to the OsFLT13 group (Clade II).

### Genetic and comparative mapping

**Figure 3:**
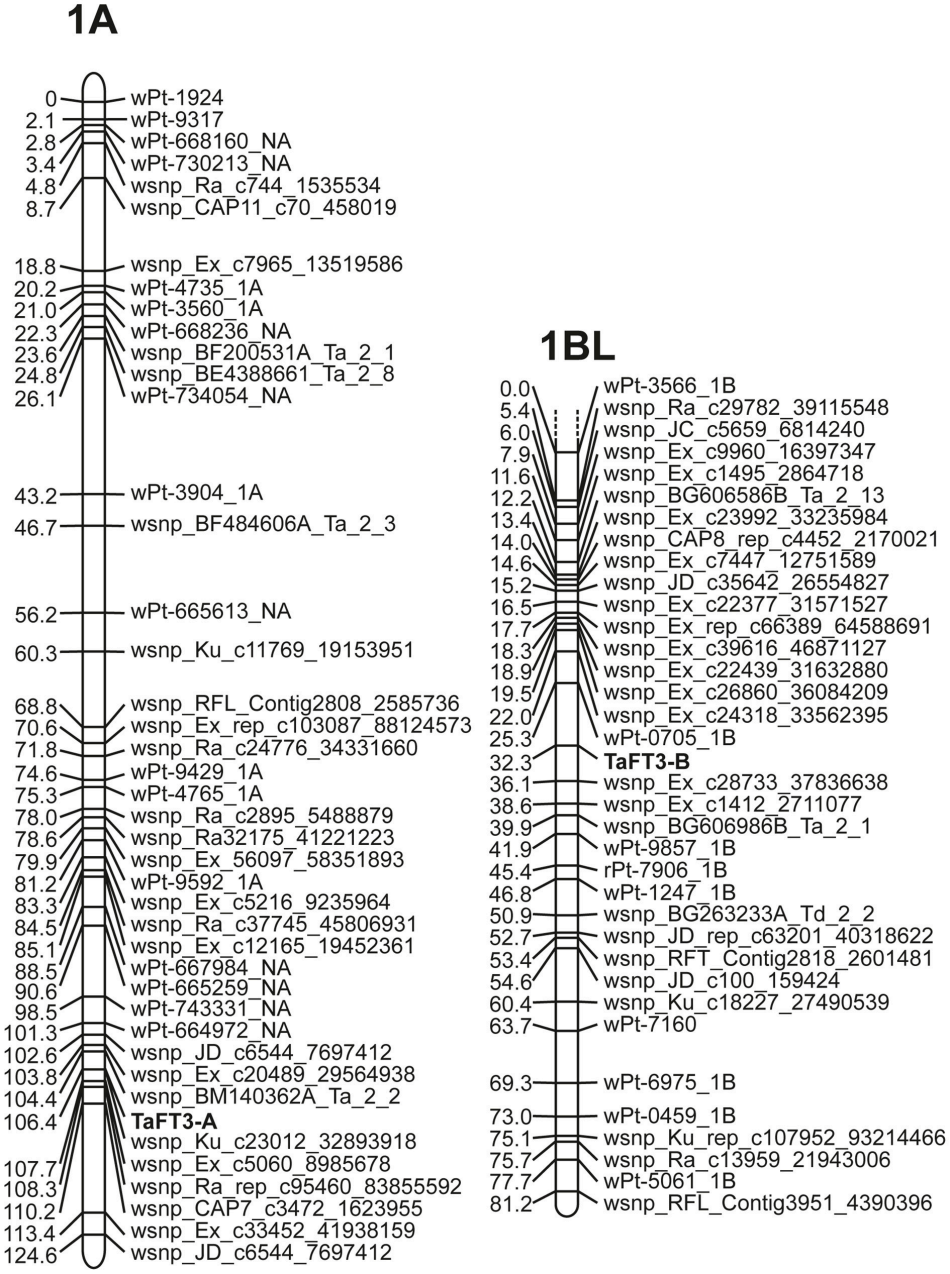
**Genetic mapping of ***TaFT3*** homoeologs to wheat chromosome 1A and the long arm of chromosome 1BL**. No polymorphism was found for *TaFT3-D1*, and so was not mapped here.

Genome-specific primers for amplification of *TaFT3-A1, TaFT3-B1*, and *TaFT3-D1* were designed from wheat genome survey sequence contigs 1AL_913428, 1BL_2932591, and 1DL_2227901, respectively (Table 1), and used for PCR amplification from the parents of a doubled haploid wheat mapping population (Robigus × Solstice, *n* = 200; Gordon et al., 2015). Genomic DNA sequences for *TaFT3* homoeologs from Robigus and Solstice were deposited in GenBank under accession numbers KX161737-KX161741. Sequencing of *TaFT3-A1* found a G/A single nucleotide polymorphism (SNP) within intron 2, 494 bp downstream of the predicted start codon, and designated G+494/A. Genotyping the DH population for this SNP allowed *TaFT3-A1* to be genetically mapped to 106.4 cM on the long arm of chromosome 1A, co-segregating with SNP wsnp_Ku_c23012_32893918 (Figure 3). Optimization of *TaFT3-B1* primers found that while PCR products of expected size (1095 bp) were amplified from Solstice, no products were obtained for Robigus. Genotyping this presence/absence polymorphism in the mapping population found *TaFT3-B1* to map to the long arm of chromosome 1B, ~32 cM from the centromere, between DArT marker wPt-0705_1B and SNP marker wsnp_Ex_c28733_37836638 (Figure 3). Using primer pairs that span all four exons of *TaFT3-D1* (TaFT3D1-F1/TaFT3D1-R1 and TaFT3D1-F4/TaFT3D1-R4) no polymorphism was detected between Robigus and Solstice, or between parents of a second mapping population (Avalon × Cadenza), and so was not genetically mapped here.

**Figure 4:**
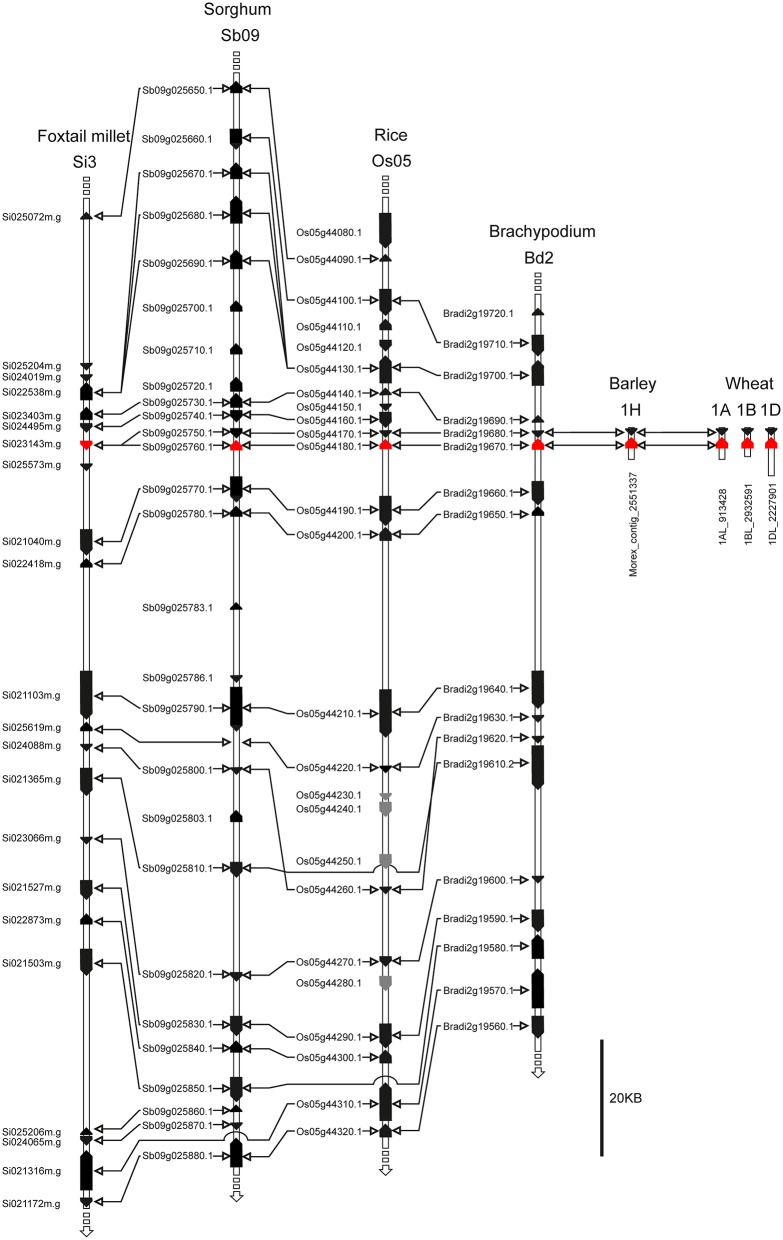
*****FT3*** region micro-colinearity between the sequenced cereal species rice, brachypodium, sorghum and foxtail millet, as well as genomic sequence contigs of barley and wheat**. Si023143m.g is identified as the sorghum ortholog of *OsFLT10*. However, for all protein-based analyses, a *de novo* gene prediction was used (Table 2, Figures 1, 2 and Supplementary Figure 1).

Macro-colinearity shows that the long arms of the wheat group 1 chromosomes are colinear with rice chromosomes Os05, brachypodium Bd2, sorghum Sb09, and foxtail millet Si3 (Devos, 2005; Paterson et al., 2009; International Brachypodium Initiative, 2010). To further investigate whether the putative *TaFT3* genes identified were orthologous to *HvFT3*, analysis of colinearity between the chromosomal locations of *FT3* genes in wheat, barley and four sequenced grass species investigated was undertaken. As expected, good conservation of macro-colinearity was observed between wheat group 1 chromosomes and rice chromosome Os05 (Supplementary Table 3). On wheat chromosome 1AL, of the 25 markers investigated, 12 identified rice orthologs on chromosome Os05 by BLASTn, positioned within a 5.7 Mbp interval between 20.9 and 26.6 Mbp. Similarly, the 36 1BL markers identified 14 rice chromosome Os05 orthologs, located within a 5.4 Mbp interval between 22.6 and 28 Mbp. *TaFT3-A1* cosegregated with wsnp_Ku_c23012_32893918, which shows high homology to genes in rice (LOC_Os05g43230; 88 gene models from *OsFTL10*), brachypodium (Bradi2g20460, 49 gene models from *BdFT3*), and sorghum (Sb09g024940, 87 gene models from *SbFT3*) (Figure 3). *TaFT3-B1* mapped between DArT marker wPt-0705 (no sequence available for comparative analysis) and SNP marker wsnp_Ex_c28733_37836638 (non-colinear homology to rice gene Os06g12400). Investigation of micro-colinearity found very good conservation of gene order within the *FT3* regions of rice, sorghum, foxtail millet and brachypodium (Figure 4), with between 78% (sorghum) and 94% (brachypodium) of all genes identifying orthologs in at least one other grass species. Colinearity extended to the available barley and wheat *FT3* genomic sequence contigs, with orthologous gene models immediately adjacent to *FT3* genes being identified in all six species.

### *TaFT3* gene expression

**Figure 5:**
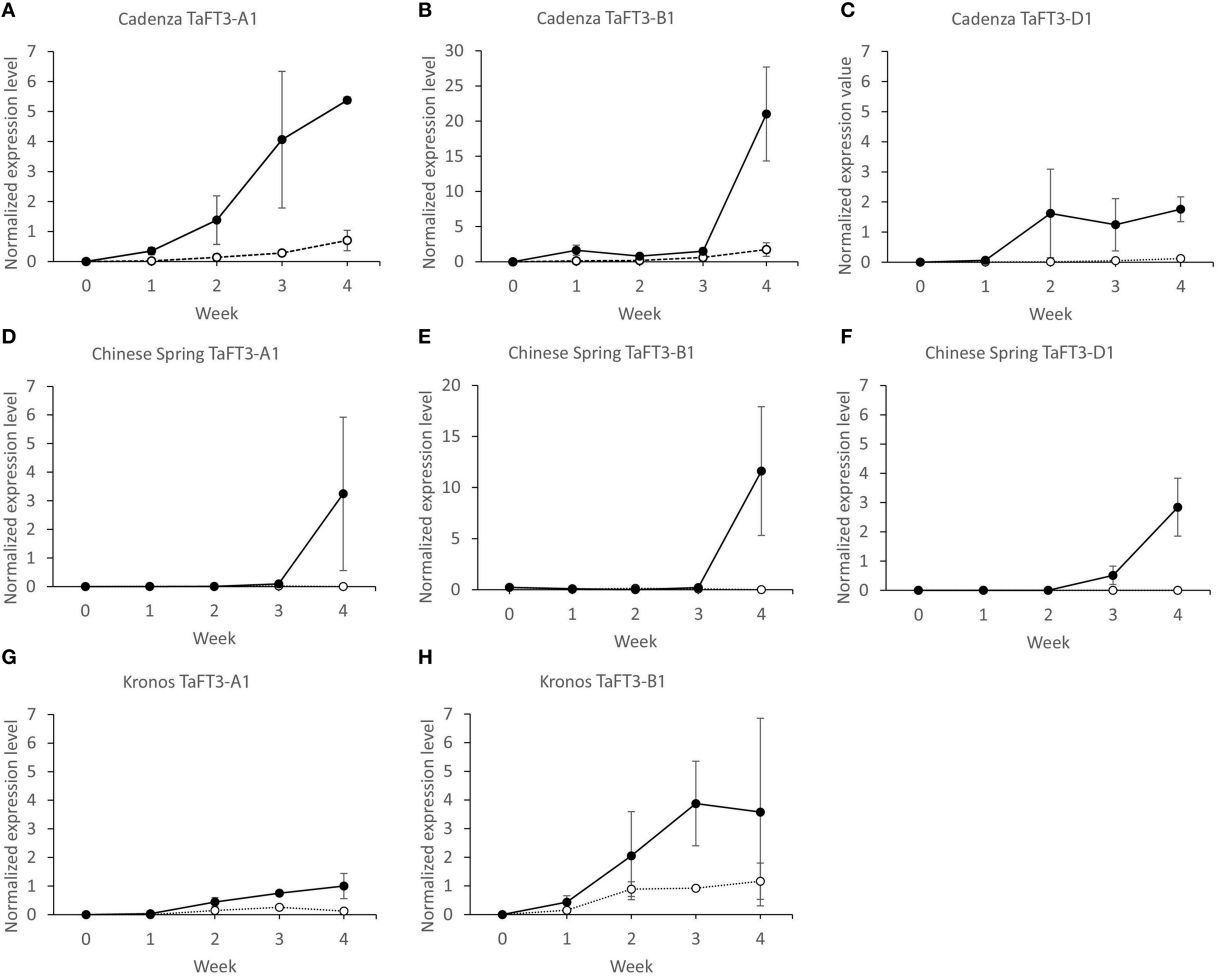
*****TaFT3*** homoeolog-specific qRT-PCR gene expression in hexaploid wheat cultivars Cadenza (A–C) and Chinese Spring (D–F) and tetraploid wheat cultivar Kronos (G,H) grown under LD (white circles) and SD (black circles) photoperiods**. Leaf samples for expression analyses were harvested at 0, 1, 2, 3 and 4 weeks. qRT-PCR *TaFT3* expression data is normalized against four control genes (*ACTIN, UBIQUITIN, GAPDH, EF1A*). ± 1 standard error of the mean (SEM) indicated.

*TaFT3* homoeolog specific expression was investigated in leaf tissue sampled at five time points (0, 1, 2, 3, and 4 weeks) for two hexaploid and one tetraploid wheat varieties grown under contrasting photoperiods, with transition from vegetative to reproductive shoot apical meristems (SAMs) found to occur between weeks 3 to 4 (Supplementary Figure 2). Under LD photoperiods, expression of *TaFT3-A1, TaFT3-B1*, and *TaFT3-D1* in the hexaploid wheat variety Cadenza (Figures 5A–C) showed similar patterns, with expression starting at low levels in week 1. Over subsequent weeks, expression remained low, but increased to a maximum at week 4, at which time *TaFT3-B1* and *TaFT3-D1* transcription was significantly higher than *TaFT3-A1*. Under SDs, expression of all three homoeologs was first detected at week 1, at comparable levels to that observed under LDs. However, by week 4, SD expression reached maximum values, that when compared to expression under LDs, represents fold increases of over 7x, 36x, and 14x for the A, B and D genome homoeologs, respectively.

In hexaploid wheat variety Chinese Spring (Figures 5D–F), *TaFT3* expression under LDs across all time points was either very low (*TaFT3-A1* and *TaFT3-B1*) or not detected at all (*TaFT3-D1*). Under SD photoperiods, expression was first detected at weeks 2 (0.01, *TaFT3-A1*), 0 (0.23, *TaFT3-B1*), and 3 (0.51, *TaFT3-D1*). For all three genomes, significant increases in transcription were then observed between weeks 3 and 4, with peak expression values of 3.24, 11.60 and 2.84 for *TaFT3-A1, TaFT3-B1*, and *TaFT3-D1*, respectively. In all cases, expression was significantly higher under SDs than LDs at week 4, with *TaFT3-B1* found to show the highest fold increase (over 80x).

In the tetraploid wheat variety Kronos (Figures 5G,H), expression was first detected at week 1, under both LD and SD photoperiods. Under LDs, *TaFT3-A1* and *TaFT3-B1* expression peaked at weeks 3 (0.26) and 4 (1.16), respectively, with significant increases observed when comparing expression at week 1 with all subsequent time points. *TaFT3-A1* expression under SDs increased significantly between weeks 1 to 3, with peak transcription observed at week 4 (1.00). At all time-points, *TaFT3-A1* expression was significantly higher under SDs than under LDs, with this difference being most pronounced at week 4 (>8x-fold increase in expression). As in the hexaploid varieties, *TaFT3-B1* expression under LDs was consistently low, reaching a peak at week 4 (1.16). Mean expression at all time-points was higher under SDs compared to LDs, and while significant differences were only observed at week 3, comparison of SD expression between week 1 and all subsequent time points found transcription to be significantly higher in all cases.

## Discussion

In Arabidopsis, *FT* is a key component in the floral signaling pathway (Kardailsky et al., 1999; Kobayashi et al., 1999). It is directly activated by CONSTANS (CO) in the leaf vasculature, after which *FT* acts as a mobile signal to promote floral transition in the apical meristem (reviewed by Shrestha et al., 2014). Members of the *FT* gene family are also known to play important roles in the control of floral transitioning and flowering in a wide range of other species (Bentley et al., 2013a), including tree species such as poplar (Böhlenius et al., 2006) and crops such as potato (Navarro et al., 2011), and onion (Lee et al., 2013). Within the Poaceae, *FT1* genes control flowering time in the SD photoperiod response species rice [*Hd3a*, (Kojima et al., 2002). *RICE FLOWERING LOCUS T1* (*RFT1*), Komiya et al., 2008] and maize [*CENTRORADIALIS 8* (*ZCN8*), Meng et al., 2011], as well as the LD species barley (*VRN-H3*), and wheat (*VRN-B3*) (Yan et al., 2006). Natural genetic variation at *HvFT3* is thought to underlie the major barley SD photoperiod response locus, *PPD-H2* (Faure et al., 2007; Kikuchi et al., 2009; Casao et al., 2011a,b; Cockram et al., 2015). Additionally, transgenic approaches have shown that silencing or upregulation of *FT1* in both brachypodium and bread wheat leads to extreme effects on flowering under both SD and LD photoperiods (Lv et al., 2014). The central role *FT* genes play in grass crop species, and the importance of flowering time to yield, mean that determining the *FT* gene families in the Poaceae has great potential for the precise manipulation of crop phenology. Here, we systematically characterize the *FT* gene families in rice, sorghum, foxtail millet, brachypodium, barley and bread wheat, identifying previously unreported members. These include homologs of rice *OsFTL4, OsFTL5*, and *OsFTL6*, as well as the members exclusive to the temperate cereals species (*FT8, FT11*). While Peng et al. (2015) identified and listed wheat and barley genes predicted to encode PEBP domains using sequence data from Arabidopsis, no systematic investigation was undertaken to compare to other *FT* family genes within or between species. Therefore, the detailed delineation of Poaceae *FT* gene families undertaken here provides a detailed baseline for future studies into this important gene family.

Copy number variation (CNV) of flowering pathway genes within Poaceae species is known to play a prominent role in the control of flowering (Cockram et al., 2010). Examples include CNVs of genes underlying the wheat major flowering time loci *PPD-B1, VRN-A1* (Díaz et al., 2012), *VRN-H3* (Nitcher et al., 2013; Loscos et al., 2014), and *VRN-D4* (Kippes et al., 2015), as well as at the barley *VRN-H2* locus, differentiating between spring and winter seasonal growth habit and influencing the spread of barley cultivation into new agricultural environments (Karsai et al., 2005; Cockram et al., 2007c). Similarly, deletion of the rice gene *Ghd7* resulted in the abolishment of delayed flowering under LD photoperiods found in tropical rice, allowing spread of cultivation to temperate regions (Xue et al., 2008). The *TaFT3-B1* presence/absence polymorphism identified here between the cultivars Robigus and Solstice indicates an intra-specific deletion event has occurred. This mirrors the presence/absence *HvFT3* polymorphism in barley that is thought to be responsible for the recessive *ppd-H2* allele, which confers delayed flowering under SD photoperiods (Faure et al., 2007; Kikuchi et al., 2009; Cockram et al., 2015). No flowering time QTL spanning the *TaFT3-B1* location has been reported in the Robigus X Solstice population (Gordon et al., 2015). This could be due to a variety of factors, including compensation by homoeologs on the A and D genomes and the lack of ability to detect appropriate QTL under the experimental conditions used. However, the identification of intra-specific *TaFT3* CNV will guide future investigations, including the possibility to harness this variant in combination with natural or artificially induced mutations at homoeologous loci to create strong variation in flowering time under SD photoperiods. In addition to this example of intra-specific variation, inter-specific CNV within the *FT* gene families was found to be relatively common between all Poaceae species investigated. This included duplication of one or more *FT* gene members (all species), lineage-specific members, as well as species specific CNV. *FT* members identified as lineage specific, or as exhibiting inter-specific CNV represent good candidates for further investigation, given the prominent role of CNV underlying known Poaceae flowering time loci. It may also be possible that *FT* members involved in inter-specific CNV are also prone to intra-specific CNV, thus highlighting a possible route toward identifying genetic variation of importance to the control of flowering. Similar examples have previously been raised, such as the lack of genes orthologous to the wheat and barley *VRN-2* loci in brachypodium, which may be due to the brachypodium accession sequenced being a mutant rapid cycling vernalization insensitive spring type (Cockram et al., 2010, 2012).

Due to the role of natural *HvFT3* variants in the control of SD photoperiod response in the diploid crop barley, and the possibility that manipulating orthologs in polyploid wheat species could lead to the creation of a new major flowering time locus, the main focus of this study was molecularly characterize orthologs in bread wheat. Multiple lines of evidence suggest we have successfully achieved this aim. Firstly, searches of the wheat genome using both *HvFT3* and the rice ortholog *OsFLT10* identified three wheat homoeologs, all of which originated from genomic DNA from the long arm of the wheat group 1 chromosomes, known to show macro-colinearity with the corresponding regions of barley chromosome 1H and rice chromosome Os05 (Faure et al., 2007; Higgins et al., 2010). Genetic mapping of *TaFT3-A1* and *TaFT3-B1* located these genes within regions broadly colinear with *OsFTL10*. While *TaFT3-D1* was not genetically mapped to the sub chromosome arm level here, previous studies investigating its candidature for an earliness *per se* (*eps*) locus have found it to map to the long arm of chromosome 1D, co-segregating with microsatellite marker *Xcfd63* and closely linked to *Xgdm111, XBarc62*, and *TaBradi2g14790* (Zikhali et al., 2014). BLASTn analysis confirms the gene termed “*TaFT3-D*” and mapped to chromosome 1DL by Zikhali et al. (2014) corresponds to the *TaFT3-D1* gene identified here (GenBank accession KJ661740 vs. contig 1DL_2227901, respectively. *E*-value = 0, percent identity 100%). Similarly, investigation of micro-colinearity around the *FT3* physical regions in six Poaceae species confirmed (i) high conservation of gene order despite the evolutionary divergence of the grasses between 55 to 70 million years ago (Kellogg, 2001), and (ii) that the Poaceae *FT3* genes identified here are truly orthologs of *OsFLT10*. Finally, we show that expression of *TaFT3* orthologs in both tetraploid and hexaploid wheat show strong upregulation under SD photoperiods during development, and that this is most pronounced at and after the transition from the vegetative to reproductive meristem stage. The conservation of SD-induced *FT3* upregulation between wheat and barley (Faure et al., 2007; Casao et al., 2011a,b) supports the assumption that wheat *TaFT3* homoeologs may play a similar role to *HvFT3* in barley, and that using reverse genetic approaches to generate *TaFT3* knock-out mutants could result in the creation of lines delayed in flowering under SDs. Such TILLING resources exist for both tetraploid (Uauy et al., 2009) and hexaploid (Rakszegi et al., 2010) wheat. Indeed, recent efforts to re-sequence TILLING lines show that it should be possible to identify mutated alleles via BLASTn searches of such TILLING lines re-sequenced using exome capture (King et al., 2015). It should be noted that the apparent absence of *TaFT3-B1* in the variety Robigus indicates that EMS and Eco-TILLING approaches could be effectively combined to stack null *TaFT3* alleles. Given the assumption that recessive null mutations analogous to the barley *ppd-H2* allele have not been identified and combined in a hexaploid bread wheat background to date, the creation of germplasm displaying delayed flowering under SDs via pyramiding of *TaFT3* homoeolog knock out mutations would be beneficial for autumn-sown wheat, as it would help delay the transition from vegetative to reproductive phase under the short day lengths experienced in the winter months (Bentley et al., 2013a). While most autumn sown cereal varieties possesses a vernalization requirement (that delays SAM transition until a prolonged period of vernalizing temperatures has been undergone) (Cockram et al., 2007c), this is likely to be satisfied by up to 8 weeks exposure. Therefore, the creation of lines where floral transition is delayed specifically under SDs would provide an additional check on floral transition until the onset of more favorable conditions in the spring, thus helping to prevent damage to delicate reproductive organs. We speculate that wheat lines with delayed in flowering under SDs conferred by mutation of *TaFT3* homologs would be best combined in autumn-sown wheat varieties, in combination with alleles at the major *PPD* and *VRN* genes previously known to best adapt the variety to relevant regional photoperiod and temperature conditions (reviewed by Bentley et al., 2013a). The sequence data and genome-specific primer pairs generated here provide the necessary tools to undertake reverse genetics approaches for the generation of lines with artificially mutated *TaFT3* alleles in wheat.

## Author contributions

JC conceived of the study, JH, PB, MP, BS, AG, RK, and JC conducted research, AB, CU, and JC provided project resources, JC, AB, PB, and CU provided project management, JH and JC wrote the manuscript, all authors reviewed the manuscript.

## Funding

JH acknowledges support by a grant from the Biotechnology and Biological Sciences Research Council (BBSRC) in support of her participation on the University of East Anglia Plant Genetics and Crop Improvement MSc course. MP was supported by an Erasmus grant under the LLP Erasmus Placement Programme. JC and AB were partially funded by BBSRC projects BB/M011666/1 and BB/I002561/1, respectively.

### Conflict of interest statement

The authors declare that the research was conducted in the absence of any commercial or financial relationships that could be construed as a potential conflict of interest.
