# Supplementary material for: Systematic Investigation of FLOWERING LOCUS T-Like Poaceae Gene Families Identifies the Short-Day Expressed Flowering Pathway Gene, TaFT3 in Wheat (Triticum aestivum L.)
Source: Front Plant Sci. 2016 Jun 22;7:857. doi: 10.3389/fpls.2016.00857 (PMC4937749; doi:10.3389/fpls.2016.00857)
Supplement: Supplementary file 1 [file Image1.PDF]

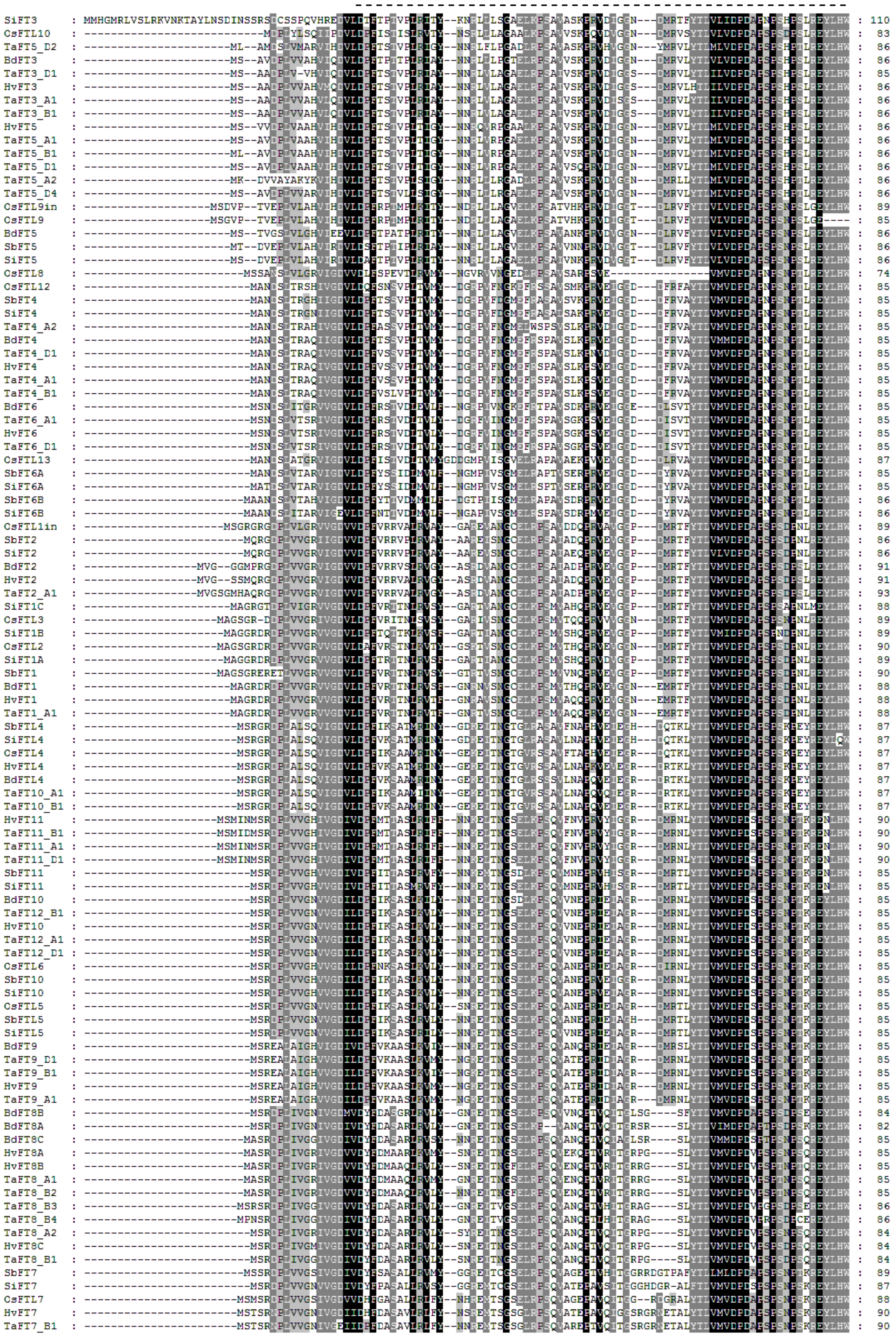

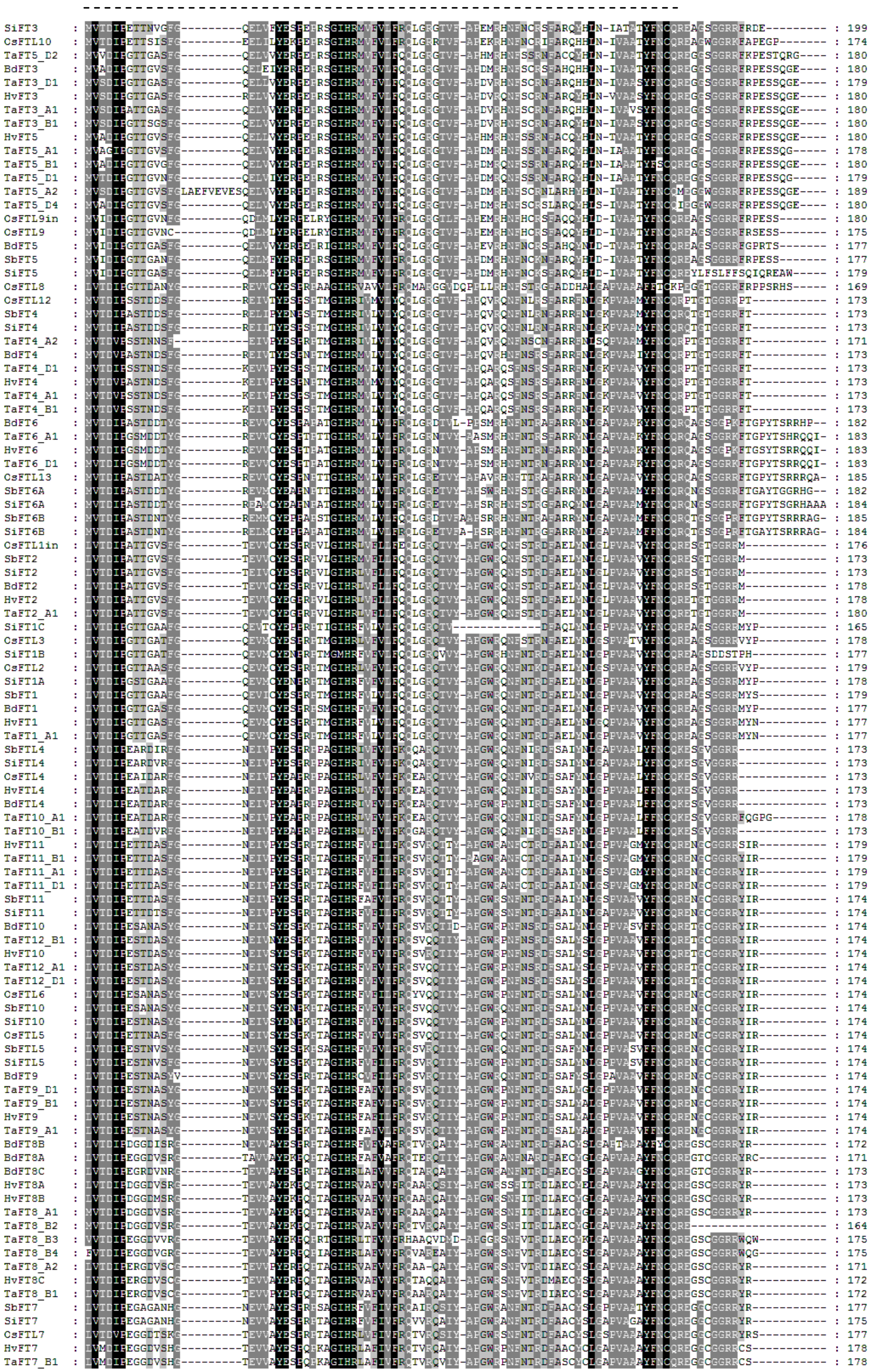

Supplementary Figure 1. Multiple alignments of 99 full-length FT predicted proteins identified in rice, brachypodium, sorghum, foxtail millet, barley and bread wheat. Accessions used are the predicted proteins from the gene models listed in Table 2. The position of the PEBP domain is indicated by the dashed line above the alignments.
