# Supplementary material for: Systematic Investigation of FLOWERING LOCUS T-Like Poaceae Gene Families Identifies the Short-Day Expressed Flowering Pathway Gene, TaFT3 in Wheat (Triticum aestivum L.)
Source: Front Plant Sci. 2016 Jun 22;7:857. doi: 10.3389/fpls.2016.00857 (PMC4937749; doi:10.3389/fpls.2016.00857)
Supplement: Supplementary file 2 [file Image2.PDF]

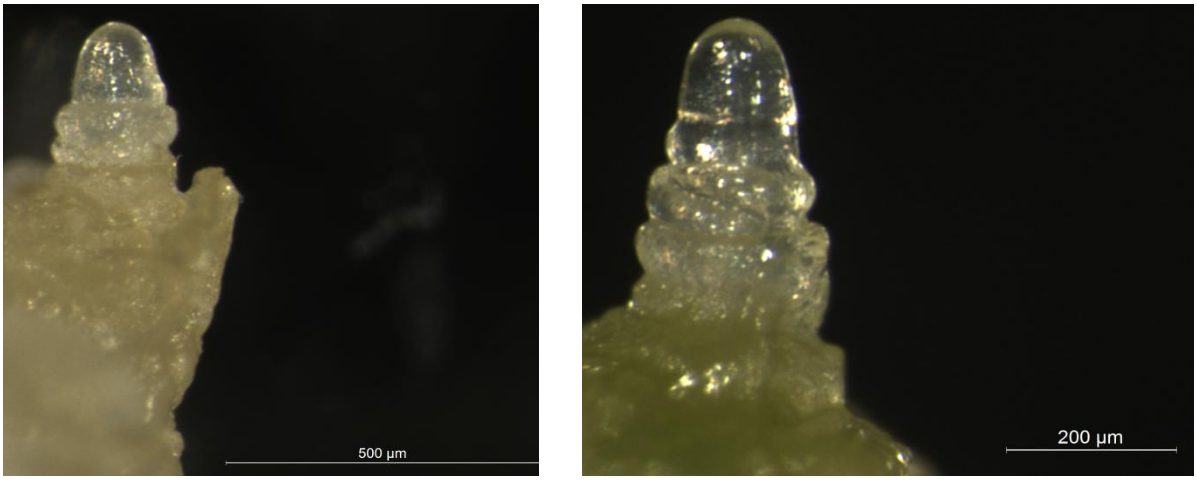

Supplementary Figure 2. Examples of meristem dissections of Chinese Spring seedling at week 3 (left) and week 4 (right) showing a transition from vegetative growth (left) to reproductive growth (right) by the presence of the double ridge.
