# Supplementary material for: Systematic Investigation of FLOWERING LOCUS T-Like Poaceae Gene Families Identifies the Short-Day Expressed Flowering Pathway Gene, TaFT3 in Wheat (Triticum aestivum L.)
Source: Front Plant Sci. 2016 Jun 22;7:857. doi: 10.3389/fpls.2016.00857 (PMC4937749; doi:10.3389/fpls.2016.00857)
Supplement: Supplementary file 3 [file Table1.DOCX]

| **Gene** | **Forward primer (5’ 🡪 3’)** | **Reverse primer (5’ 🡪 3’)** |
| --- | --- | --- |
| *ACTIN* | ACCTTCAGTTGCCCAGCAAT | CAGAGTCGAGCACAATACCAGTTG |
| *UBIQUITIN* | CCTTCACTTGGTTCTCCGTCT | AACGACCAGGACGACAGACACA |
| *GAPDH* | TTAGACTTGCGAAGCCAGCA | AAATGCCCTTGAGGTTTCCC |
| *EF1A* | TGGTGTCATCAAGCCTGGTATGGT | ACTCATGGTGCATCTCAACGGACT |

**Supplementary Table 1.** Primer details of control genes used for qRT-PCR.
