# Supplementary material for: Systematic Investigation of FLOWERING LOCUS T-Like Poaceae Gene Families Identifies the Short-Day Expressed Flowering Pathway Gene, TaFT3 in Wheat (Triticum aestivum L.)
Source: Front Plant Sci. 2016 Jun 22;7:857. doi: 10.3389/fpls.2016.00857 (PMC4937749; doi:10.3389/fpls.2016.00857)
Supplement: Supplementary file 4 [file Table2.DOCX]

| **Wheat contig** | **e-value^*^** | **Chr** | **Contig length (bp)** | ***TaFT3* CDS (exons)** | **TaFT3 protein (aa)** | **Protein domain (e-value)** |
| --- | --- | --- | --- | --- | --- | --- |
| 1AL_913428 | 1e-107 | 1AL | 3,801 | 543 bp (4) | 180 | PBP (2.3e-21) |
| 1BL_2932591 | 1e-106 | 1BL | 3,752 | 543 bp (4) | 180 | PBP (2.6e-21) |
| 1DL_2227901 | 3e-109 | 1DL | 13,083 | 540 bp (4) | 179 | PBP (2e-21) |

**Supplementary Table 2.** Wheat genomic sequence hits on the group 1 chromosomes using *HvFT3* CDS as the query. Chr = chromosome. CDS = coding regions. PBP = Phosphatidylethanolamine-binding protein. ^*^ *HvFT3* CDS versus wheat.
