# Supplementary material for: Systematic Investigation of FLOWERING LOCUS T-Like Poaceae Gene Families Identifies the Short-Day Expressed Flowering Pathway Gene, TaFT3 in Wheat (Triticum aestivum L.)
Source: Front Plant Sci. 2016 Jun 22;7:857. doi: 10.3389/fpls.2016.00857 (PMC4937749; doi:10.3389/fpls.2016.00857)
Supplement: Supplementary file 5 [file Table3.docx]

| **Wheat chromosome 1AL marker** | **Wheat chromosome 1A cM position** | **Rice gene model** | **Rice chromosome Os05 Mbp position** |
| --- | --- | --- | --- |
| wsnp_RFL_Contig2808_2585736 | 66.8 | LOC_Os10g35720.1 |  |
| wsnp_Ex_rep_c103087_88124573 | 70.6 | LOC_Os10g40110.1 |  |
| wsnp_Ra_c24776_34331660 | 71.8 | no hit |  |
| wPt-9429 | 74.6 | NSA |  |
| wPt-4765 | 75.3 | NSA |  |
| wsnp_Ra_c2895_5488879 | 78 | LOC_Os05g35274.2 | 20.9 |
| wsnp_Ra_c32175_41221223 | 78.6 | LOC_Os05g35710.1 | 21.2 |
| wsnp_Ex_c56097_58351893 | 79.9 | LOC_Os05g37350.1 | 21.8 |
| wPt-9592_ | 81.2 | NSA |  |
| wsnp_Ex_c5216_9235964 | 83.3 | LOC_Os05g38460.1 | 22.6 |
| wsnp_Ra_c37745_45806931 | 84.5 | LOC_Os05g38810.1 | 22.8 |
| wsnp_Ex_c12165_19452361 | 85.1 | LOC_Os05g39080.1 | 22.9 |
| wPt-667984_NA | 88.5 | NSA |  |
| wPt-665259_NA | 90.6 | NSA |  |
| wPt-743331_NA | 98.5 | NSA |  |
| wPt-664972_NA | 101.3 | NSA |  |
| wsnp_JD_c6544_7697412 | 102.6 | no hit |  |
| wsnp_Ex_c20489_29564938 | 103.8 | LOC_Os05g41930.1 | 24.6 |
| wsnp_BM140362A_Ta_2_2 | 104.4 | no hit |  |
| **TaFT3-A** | **106.4** | **LOC_Os05g44180** | **25.7** |
| wsnp_Ku_c23012_32893918 | 107.7 | LOC_Os05g43230.1 | 25.2 |
| wsnp_Ex_c5060_8985678 | 108.3 | LOC_Os08g30740.1 |  |
| wsnp_Ra_rep_c95460_83855592 | 110.2 | LOC_Os05g44916.1 | 26.1 |
| wsnp_CAP7_c3472_1623955 | 113.4 | LOC_Os05g45310.1 | 26.3 |
| wsnp_Ex_c33452_41938159 | 124.6 | LOC_Os05g45930.1 | 26.6 |
| **Wheat chromosome 1BL marker** | **Wheat chromosome 1BL cM position** | **Rice gene model** | **Rice chromosome Os05 Mbp position** |
| wPt-3566_1B | 0 |  |  |
| wsnp_Ra_c29782_39115548 | 5.4 | LOC_Os05g38460.1 | 22.6 |
| wsnp_JD_c5659_6814240 | 6 | LOC_Os05g38370.1 | 22.5 |
| wsnp_Ex_c9960_16397347 | 7.9 | LOC_Os05g34760.1 | 20.6 |
| wsnp_Ex_c1495_2864718 | 11.6 | no hit |  |
| wsnp_BG606586B_Ta_2_13 | 12.2 | no hit |  |
| wsnp_Ex_c23992_33235984 | 13.4 | LOC_Os05g39850.1 | 23.4 |
| wsnp_CAP8_rep_c4452_2170021 | 14 | LOC_Os01g41710.1 |  |
| wsnp_Ex_c7447_12751589 | 14.6 | LOC_Os05g41150.1 | 24.1 |
| wsnp_JD_c35642_26554827 | 15.2 | no hit |  |
| wsnp_Ex_c22377_31571527 | 16.5 | no hit |  |
| wsnp_Ex_rep_c66389_64588691 | 17.7 | NSA |  |
| wsnp_Ex_c39616_46871127 | 18.3 | LOC_Os05g42300.1 | 24.7 |
| wsnp_Ex_c22439_31632880 | 18.9 | LOC_Os05g42350.1 | 24.8 |
| wsnp_Ex_c26860_36084209 | 19.5 | NSA |  |
| wsnp_Ex_c24318_33562395 | 22 | LOC_Os05g43610.1 | 25.4 |
| wPt-0705_1B | 25.3 | no hit |  |
| **TaFT3-B** | **32.3** | **LOC_Os05g44180** | **25.7** |
| wsnp_Ex_c28733_37836638 | 36.1 | LOC_Os06g12400.1 |  |
| wsnp_Ex_c1412_2711077 | 38.6 | LOC_Os11g42800.1 |  |
| wsnp_BG606986B_Ta_2_1 | 39.9 | LOC_Os05g45420.1 | 26.3 |
| wPt-9857_1B | 41.9 | NSA |  |
| rPt-7906_1B | 45.4 | NSA |  |
| wPt-1247_1B | 46.8 | NSA |  |
| wsnp_BG263233A_Td_2_2 | 50.9 | NSA |  |
| wsnp_JD_rep_c63201_40318622 | 52.7 | LOC_Os02g57540.1 |  |
| wsnp_RFL_Contig2818_2601481 | 53.4 | LOC_Os05g47550.1 | 27.2 |
| wsnp_JD_c100_159424 | 54.6 | no hit |  |
| wsnp_Ku_c18227_27490539 | 60.4 | LOC_Os05g47780.1 | 27.4 |
| wPt-7160 | 63.7 | NSA |  |
| wPt-6975_1B | 69.3 | NSA |  |
| wPt-0459_1B | 73 | NSA |  |
| wsnp_Ku_rep_c107952_93214466 | 75.1 | LOC_Os05g48600.1 | 27.9 |
| wsnp_Ra_c13959_21943006 | 75.7 | NSA |  |
| wPt-5061_1B | 77.7 | NSA |  |
| wsnp_RFL_Contig3951_4390396 | 81.2 | LOC_Os05g48800.1 | 28.0 |

**Supplementary Table 3.** Colinearity between wheat group 1 chromosomes and rice, based on genetic and physical maps, respectively. NSA = no sequence available. NA = not available.
